# Recent Advances in the Diagnosis and Treatment of Natural Killer Cell Malignancies

**DOI:** 10.3390/cancers14030597

**Published:** 2022-01-25

**Authors:** Eric Tse, Yok-Lam Kwong

**Affiliations:** Department of Medicine, Queen Mary Hospital, Hong Kong, China; ewctse@hku.hk

**Keywords:** NK/T-cell lymphomas, EBV, asparaginase, radiotherapy, haematopoietic stem cell transplantation, PD1

## Abstract

**Simple Summary:**

Natural killer (NK)/T-cell lymphomas are aggressive extranodal Epstein–Barr virus (EBV)-positive malignancies. They can be divided into three subtypes: nasal (involving the nose and upper aerodigestive tract), non-nasal (involving skin, gastrointestinal tract, testis and other organs) and disseminated (involving multiple organs). Lymphoma cells are positive for CD3ε, CD56, cytotoxic molecules and EBV-encoded small RNA. There is a predilection for Asian and Central/South American populations. Genome-wide association studies have identified lymphoma susceptibility loci in Asians. Positron emission tomography computed tomography and plasma EBV DNA quantification are crucial at diagnosis and follow-up. Stage I/II patients receive non-athracycline asparaginse-containing regimens, together with sequential/concurrent radiotherapy. Anthracycline-containing regimens are ineffective. Stage III/IV patients receive asparaginase-containing regimens, followed by allogeneic haematopoietic stem cell transplantation (HSCT). Autologous HSCT does not improve outcome. In relapsed/refractory patients, novel approaches include PD1/PD-L1 targeting, EBV-specific cytotoxic T-cells, and monoclonal antibodies. Small molecules including histone deacetylase inhibitors may be beneficial.

**Abstract:**

Natural killer (NK)/T-cell lymphomas are aggressive malignancies. Epstein–Barr virus (EBV) infection in lymphoma cells is invariable. NK/T-cell lymphomas are divided into nasal, non-nasal, and disseminated subtypes. Nasal NK/T-cell lymphomas involve the nasal cavity and the upper aerodigestive tract. Non-nasal NK/T-cell lymphomas involve the skin, gastrointestinal tract, testis and other extranodal sites. Disseminated NK/T-cell lymphoma involves multiple organs, rarely presenting with a leukaemic phase. Lymphoma cells are positive for CD3ε (not surface CD3), CD56, cytotoxic molecules and EBV-encoded small RNA. There is a predilection for Asian and Central/South American populations. Genome-wide association studies have identified lymphoma susceptibility loci in Asian patients. Positron emission tomography computed tomography and plasma EBV DNA quantification are crucial evaluations at diagnosis and follow-up. Stage I/II patients typically receive non-athracycline regimens containing asparaginse, together with sequential/concurrent radiotherapy. Anthracycline-containing regimens are ineffective. Stage III/IV patients are treated with asparaginase-containing regimens, followed by allogeneic haematopoietic stem cell transplantation (HSCT) in suitable cases. Autologous HSCT does not improve outcome. In relapsed/refractory patients, novel approaches are needed, involving PD1/PD-L1 targeting, EBV-specific cytotoxic T-cells, and monoclonal antibodies. Small molecules including histone deacetylase inhibitors may be beneficial in selected patients. Future strategies may include targeting of signalling pathways and driver mutations.

## 1. Introduction

Natural killer (NK) cells develop from a common lymphoid progenitor capable of differentiation to all lymphoid lineages [1]. Expression of the transcription regulators STAT5, NFIL3, NOTCH, PU.1, TCF1, RUNX3 and CBFB leads to NK lineage commitment [2]. NK-cells are part of the innate immune system and are responsible for detection and killing of tumour or virally infected cells [3]. They are strategically located at mucosal surfaces, the skin and gut, and they circulate between the blood and bone marrow [3]. The normal residency of NK-cells in these sites accounts for the predominant extranodal localization of malignancies arising from neoplastic NK-cells [4]. NK-cells express CD2, cytoplasmic CD3 epsilon (ε) (not surface CD3), CD16, CD56, CD94, and cytotoxic molecules (perforin, granzyme, T-cell intracellular antigen 1, TIA-1). Immunophenotypically, this pattern of antigen expression is preserved in NK-cell malignancies [4].

First reported more than seven decades ago, lethal midline granuloma was described as destructive midline facial malignancies that resulted inexorably in death [5]. Histologically, these lesions showed neoplastic lymphoid cells admixed with inflammatory cells and were morphologically referred to as polymorphic reticulosis. The initial detection of cytoplasmic CD3ε, together with the angiocentric and angiodestructive features of the tumour, resulted in its classification as an angiocentric T-cell lymphoma [6].

Further refinement of immunophenotyping revealed that these angiocentric T-cell lymphomas were in most cases surface CD3 negative, with T-cell receptor (*TCR*) genes in a germline configuration. Therefore, lymphoma cells showed feature typical of NK-cells. However, cases with similar anatomical localization and pathologic features may be of bona fide T-cell origin, expressing surface CD3 and possessing clonal *TCR* gene rearrangement [7]. Whether of putative NK-cell or T-cell derivations, these cases have indistinguishable clinicopathologic features and comparable treatment outcome. The nomenclature extranodal NK/T-cell lymphoma is currently adopted by the World Health Organization (WHO) classification to recognize their possible NK-cell and T-cell origins [8].

## 2. Clinical Subtypes of NK/T-Cell Lymphomas

**Figure 1:**
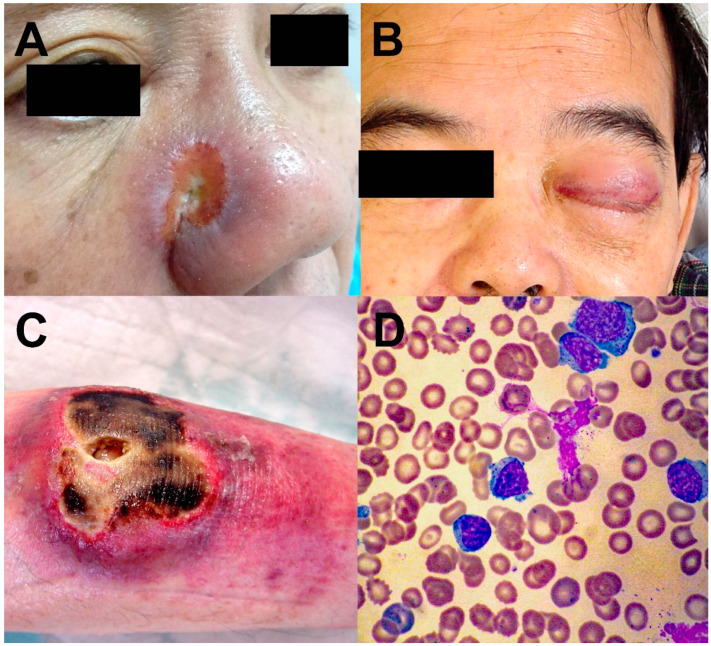
Different clinical forms of NK/T-cell lymphomas. (**A**) Nasal NK/T-cell lymphoma, eroding from the nasal cavity into the skin. (**B**) Nasal NK/T-cell lymphoma eroding into the orbit and cavernous sinus, resulting in third nerve palsy and complete ptosis. (**C**) Non-nasal NK/T-cell lymphoma of the skin. (**D**) Aggressive NK/T-cell leukaemia/lymphoma. Circulating lymphoma cells were cytologically large granular lymphocytes.

NK/T-cell lymphomas are almost exclusively extranodal in their distribution, with the nasal cavity, nasopharynx, oropharynx, the Waldeyer’s ring, and the upper aerodigestive tract most commonly involved (Figure 1A,B). Clinically, they are referred to as nasal NK/T-cell lymphomas [9]. In about 10–20% of cases, other sites, including skin, testicles, gastrointestinal tract and salivary glands, are involved. Clinically, they are referred to as the non-nasal subtype (Figure 1C). Notably, the sites involved in non-nasal NK/T-cell lymphomas are also those that nasal NK/T-cell lymphomas disseminate to in advanced stages. Hence, for non-nasal NK/T-cell lymphomas, radiologic and/or histologic investigations ought to be performed to rule out nasal involvement. For an apparent non-nasal NK/T-cell lymphoma that can be shown to have nasal involvement, the case should be reclassified as disseminated nasal NK/T-cell lymphoma [9]. Rarely, the lymphoma is disseminated on presentation and may be associated with a leukaemic phase. Clinically, they are referred to as the aggressive leukaemia/lymphoma subtype (Figure 1D) [9].

These subtypes differ in clinical presentations. However, their underlying pathology and molecular alterations are comparable such that treatment strategies for these subtypes are similar.

## 3. Pathological Features of NK/T-Cell Lymphomas

The lymphomatous infiltrate is polymorphic, with median-sized malignant lymphoid cells intermixed with inflammatory cells. Cytologically, lymphoma cells show abundant cytoplasm with azurophilic granules, resembling large granular lymphocytes. Angiocentricity and angiodestruction may be found if blood vessels are included in the biopsy, explaining the frequent occurrence of coagulative necrosis. Lymphoma cells are typically CD2+, surface CD3−, cytoplasmic CD3ε+, CD56+, and cytotoxic molecules (perforin, granzyme, TIA1)+. Epstein Barr virus (EBV) is invariably present in an episomal form [4,8]. The current WHO classification requires presence of EBV for the diagnosis of NK/T-cell lymphoma [8]. Furthermore, there should also be expression of either CD56 or cytotoxic molecules. If CD56 and cytotoxic molecules are absent, the diagnosis becomes an EBV-positive peripheral T-cell lymphoma not otherwise specified [8].

Recently, exceptional cases of EBV-negative aggressive leukaemia/lymphoma of putative NK-cell derivation had been reported [10]. However, only few cases were described; thus, it remains unclear if they were biologically or clinically similar to NK/T-cell lymphoma.

## 4. Epidemiology and Genetic Susceptibilities

NK/T-cell lymphomas occur predominantly in the Asian and Central/South American populations [4,8,9]. Genome-wide association studies in Asian patients have identified three genetic loci, *HLA-DPB1*, *IL18RAP* and *HLA-DRB1*, in which amino-acid differences may increase the susceptibility to NK/T-cell lymphoma [11,12]. HLA-DPB1 is the β1 subunit of the HLA-DP heterodimer, which participates in extracellular antigen presentation to CD4+ T-cells [11]. IL18RAP (interleukin-18 receptor associated protein) is an accessory subunit of the heterodimeric IL-18 receptor. It increases the affinity of IL-18 receptor binding to IL-18, which results in enhanced cell-mediated immunity. IL18-RAP mutations are associated with Crohn’s disease [13], and susceptibility to coeliac disease and leprosy. HLA-DRB1 is the most prevalent β subunit of the HLA-DR heterodimer. Changes in HLA-DRB1 are associated with rheumatoid arthritis [14] and increased risks of nasopharyngeal carcinoma [12]. The changes in HLA-DRB1 in NK/T-cell lymphomas are predicted to alter the peptide-binding pocket 7 of HLA-DR [12]. The association of proteins involved in immune response with susceptibility to NK/T-cell lymphoma suggests that the racial predilection may be related to genetic differences in immune reaction against EBV infection. It remains to be defined if these genetic susceptibilities may be demonstrated in other ethnic populations at risk of NK/T-cell lymphomas.

## 5. Molecular Pathology of NK/T-Cell Lymphomas

In NK/T lymphoma cells, EBV infection shows a latency II pattern, with neoplastic cells expressing LMP1, LMP2 and EBNA1 [15]. EBV is present in a clonal form, suggesting that infection occurs before malignant transformation. Furthermore, LMP1, LMP2 and EBNA1 are oncoproteins, and may contribute to lymphomagenesis.

Early karyotypic studies showed that deletion of the long-arm of chromosome 6 (6q-) was the most consistent aberration observed [16], which was confirmed by comparative genomic hybridization [17] and loss of heterozygosity analyses [18]. Putative tumour suppressor genes in this segment of chromosome 6q that might be involved in NK/T-cell lymphomagenesis include *HACE1* [19], *PRMD1* [20], *FOXO3* [21] and *PTPRK* [22]. In addition to inactivation/deletion of tumour suppressor genes, activation of putative oncogenes, including *EZH2* [23] and *RUNX3* [24], might also be involved. With gene expression profiling, other oncogenic mechanisms were also shown to be involved, including JAK/STAT and aurora kinase A activation [25] and overexpression of MYC and NF-κB [26].

**Figure 2:**
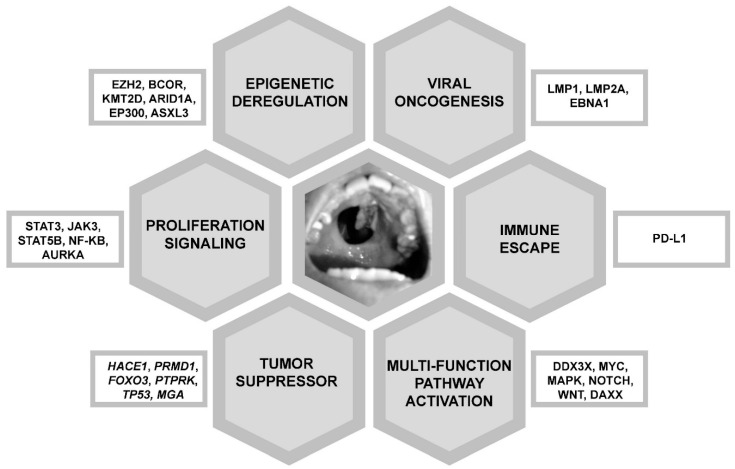
Molecular pathogenesis of NK/T-cell lymphomas. These molecular pathways are targetable therapeutically. Shown in the centre is a typical hard palate perforation due to erosion from an NK/T-cell lymphoma in the nasal cavity, giving the classical appearance of a “lethal midline granuloma”.

Next generation sequencing (NGS) of NK/T-cell lymphoma samples from various patient populations had shown different results. Mutated genes defined by NGS included *JAK3* [27], *STAT3* and *STAT5B* (proliferation signalling) [28,29]; *BCOR*, *KMT2D*, *ARID1A*, *EP300* and *ASXL3* (epigenetic deregulation) [29,30]; *TP53*, *MGA* (tumour suppressor) [30], and *DDX3X* (RNA helicase, multiple functions) [30]. These gene mutations were predicted to have different oncogenic effects (Figure 2).

Besides mutations, other mechanisms have been proposed to critically affect gene functions in NK/T-cell lymphomas. Promoter hypermethylation of *BIM*, *DAPK1*, *SHP1*, *TET2* and *SOCS6* resulted in down-regulation of these tumour suppressor genes [31]. Down-regulation of microRNAs miR-101, miR-26, miR-146a, miR-28-5 and miR-363 was predicted to result in deregulation of cell cycle-related, TP53 and MAPK signalling pathways [32]. These non-mutational mechanisms may contribute to NK/T-cell lymphomagenesis.

Overexpression of programmed cell death protein 1 ligand (PD-L1) had been shown to be a potential mechanism of immune evasion in EBV-infected lymphoid cells [33]. PD-L1 is the cognate ligand of the immune checkpoint protein PD1 expressed on T-cells. In NK/T-cell lymphomas, PD-L1 is overexpressed through a number of mechanisms [34]. The EBV oncoprotein LMP1 drives PD-L1 expression through the NF-κB pathway [35]. Furthermore, LMP1 positively regulates the expression of the transcription factor AP-1, which up-regulates PD-L1 [36]. Finally, the JAK/STAT pathway is activated in NK/T-cell lymphoma cells, which interacts with the interferon-stimulated response element located in the promoter region of PD-L1, driving its expression [37]. By binding to PD1 on T-cells and inhibiting their functions, PD-L1 enables NK/T lymphoma cells to escape immune detection, thereby enhancing their proliferation.

A combined genomic and transcriptomic approach has divided NK/T lymphoma cells into three molecular subtypes, TSIM, MB and HEA [38]. The TSIM subtype, accounting for about 55% of cases, was defined by mutations in the JAK-STAT pathway and *TP53*, amplifications of the 9p24.1/*JAK2*, 17q21.2/*STAT3/5B/5A* and 9p24.1/*PD-L1/2* loci, and deletion of chromosome 6q21. Lymphoma cells in the TSIM subtype had a predominant NK-cell gene expression pattern, activation of the JAK/STAT pathway, overexpression of PD-L1/2 and genomic instability. The MB subtype, accounting for about 18% of cases, was defined by *MGA* mutation and loss of heterozygosity (LOH) of the 1p22.1/*BRDT* locus. Lymphoma cells in the MB subtype had a gene expression pattern intermediate between NK-cells and T-cells. Mutations in *MGA* led to increased MYC expression, and together with *BRDT* LOH resulted in MAPK, NOTCH and WNT pathway activations. The HEA subtype, accounting for about 27% of cases, was defined by mutations in *HDAC9*, *EP300* and *ARID1A*. Lymphoma cells in the HES subtype had a predominant T-cell gene expression pattern, associated with overexpression of the histone chaperone DAXX, and activation of the NF-κB and T-cell receptor signalling pathways. From RNA-seq and immunohistochemical results, the TSIM, MB and HEA subtypes were characterized by overexpression of PD-L1, MYC and DAXX respectively. These findings might be of therapeutic implications.

## 6. Disease Presentations

Patients with nasal NK/T-cell lymphomas often have a long history of nasal blockage and discharge. On presentation, some destruction of nasal and facial structures is common. Nasal tumours can erode and destroy the hard palate, creating a communication between the nasal and oral cavities (the typical lethal midline granuloma), severely affecting phonation and ingestion. Invasion of the orbits may occur, affecting vision. In advanced nasal NK/T-cell lymphomas, systemic dissemination with a predilection for non-nodal organs occurs. In non-nasal NK/T-cell lymphomas, skin is the most common initial site of presentation [39]. However, virtually any anatomical sites can be involved. Interestingly, central nervous system (CNS) involvement on presentation is uncommon and usually occurs in advanced stages or relapsed disease [40].

Aggressive NK-cell leukaemia/lymphoma is currently referred to by WHO classification as aggressive NK-cell leukaemia [41]. Clinical manifestations include fever, lymphadenopathy, hepatosplenomegaly, pancytopenia, skin infiltration, deranged liver function, coagulopathy, hyperferritinaemia, and haemophagocytosis in the marrow and other reticuloendothelial organs [42]. Aggressive NK-cell leukaemia/lymphoma may arise de novo or may represent a terminal event in nasal or non-nasal NK/T-cell lymphomas.

## 7. Differential Diagnoses of NK/T-Cell Lymphomas

Plasmacytoid dendritic cell neoplasms, previously erroneously referred to as blastoid NK-cell lymphomas, are primarily a cutaneous neoplasm, although marrow infiltration occasionally occurs [43]. It can be differentiated from cutaneous NK/T-cell lymphomas by absence of CD3/CD3ε, cytotoxic molecules, and EBV infection. NK-cell lymphomatoid gastropathy/NK-cell enteropathy is a rare non-neoplastic NK-cell proliferation in the stomach, small and large bowels [44,45]. There is no EBV infection, and the disease is localized and often self-regressing. Chronic lymphoproliferative disorder of NK-cells is a rare condition, currently unclear if reactive or neoplastic. There is no EBV infection. The disease is indolent and may self-regress. No treatment is required.

## 8. Evaluation of Newly Diagnosed NK/T-Cell Lymphoma Patients

Histologic proof of organ involvement may not be straightforward, as the lymphoma infiltrate tends to be polymorphic, which is different from conventional lymphomas where a monomorphic infiltrate is found. Morphological evaluation can be supplemented by in situ hybridization for EBV encoded small RNA (EBER), which provides an accurate way of localizing lymphoma cells [8].

As lymphoma cells undergo apoptosis, fragments of EBV DNA are released into the circulation. Quantification of circulating EBV DNA therefore provides a molecular means of measuring tumour load [46]. Whole blood should not be used, because of the variable presence of circulating memory B-cells that may be infected by EBV, which introduces unpredictable errors [46]. Instead, plasma EBV DNA provides a more reliable assessment [46,47]. Plasma EBV DNA at diagnosis gives an accurate measurement of lymphoma load [48]. During treatment, plasma EBV DNA also provides a dynamic assessment of disease load, reflecting how well the lymphoma is responding to therapy [49]. Accordingly, on completion of treatment, quantifiable circulating EBV DNA indicates residual lymphoma and portends an unfavourable prognosis [50].

Different modalities of radiological examination have been adopted. NK/T-cell lymphoma cells are fluorine-18 fluorodeoxyglucose (FDG)-avid. Therefore, FDG positron emission tomography computed tomography (PET/CT) is the standard imaging modality for NK/T-cell lymphoma [51,52]. All patients should be evaluated with PET/CT on initial presentation for accurate staging. Furthermore, the five-point Deauville score should be adopted for the evaluation of PET/CT performed during and after completion of treatment [50,53].

## 9. Principles of Management of NK/T-Cell Lymphomas

Of all lymphoid cells, NK-cells express the highest level of P-glycoprotein, which confers a multidrug resistance (MDR) phenotype [54]. Anthracycline-containing (CHOP, cyclophosphamide, adriamycin, vincristine, prednisolone, or CHOP-like) regimens, designed for the treatment of conventional high-grade B-cell lymphomas, are MDR-dependent and hence ineffective [9]. Non-anthracycline-containing regimens have been developed for NK/T-cell lymphomas [9]. An important component of these regimens is asparaginase, which induces apoptosis of NK-cells in vitro [55]. Asparaginase has significant single-agent activity in relapsed/refractory (R/R) NK/T-cell lymphoma [9]. Currently, most recommended regimens for NK/T-cell lymphomas contain asparaginase or its pegylated form.

Prognostication and risk stratification are required for delivery of optimal therapy. Models developed in patients treated with CHOP or CHOP-like regimens, including the international prognostic index [56] and the Korean prognostic index [57], still retain prognostic value in patients treated with non-anthracycline regimens but have become somewhat obsolete in comparison with models that include biologic parameters. Two prognostic indices, prognostic index for NK/T-cell lymphoma (PINK) and PINK with EBV DNA (PINK-E), have been developed specifically for patients treated with non-anthracycline-containing regimens [58]. These prognostic models are based on presentation parameters. PINK includes only clinical parameters (age > 60 years, stage III/IV disease, distant lymph-node involvement, non-nasal type disease), whereas PINK-E considers additionally presentation EBV DNA (whether or not detectable), thereby incorporating one more important biologic prognosticator [48,49].

Prognostication based on dynamic parameters has also been proposed. Interim quantification of plasma EBV DNA and five-point Deauville scoring with PET/CT during treatment may predict outcome [49,53]. A combination of plasma EBV DNA quantification and PET/CT Deauville scoring at the end of treatment was also reported to be prognostic [50]. Such prognostic models will have to be validated in larger prospective studies.

### 9.1. Management of Stage I/II Disease

The treatment modalities for stage I/II NK/T-cell lymphomas include involved-field radiotherapy, chemotherapy or their combinations [59]. NK/T-cell lymphoma is radiosensitive, such that radiotherapy is conventionally considered a component of treatment. However, consensus lacks on how and when radiotherapy and chemotherapy should be used, viz., combined or sequenced [59]. This is because NK/T-cell lymphoma is uncommon even in countries where it is considered more prevalent such that large prospective phase III randomized trials to examine and compare different treatment approaches have not been conducted.

#### 9.1.1. Concurrent Chemoradiotherapy

Concurrent chemoradiotherapy is predicated on augmented radiosensitivity when chemotherapy is given at the same time. Concurrent chemoradiotherapy with the DeVIC (dexamethasone, etoposide, ifosfamide and carboplatin) [60,61], VIPD (etoposide, ifosfamide, cisplatin and dexamethasone) [62], and VIDL (etoposide, ifosfamide, dexamethasone and L-asparaginase) [63] regimens and radiotherapy (40–50 Gy) in stage I/II patients gave overall response rates (ORR) of 78–90%, and complete remission rates (CR) of 75–87%. Five-year progression-free survivals (PFS) of 60–67% and overall survivals (OS) of 72–73% were achieved. Concurrent chemoradiotherapy is logistically difficult because radiotherapy is not always immediately available in most centres. Furthermore, the concomitant use of chemotherapy and radiotherapy has significant mucosal toxicity and may not be tolerated in older patients and those with poor performance status.

#### 9.1.2. Sequential Chemotherapy and Radiotherapy

Sequential chemotherapy and radiotherapy involve delivering chemotherapy first, followed by interim or end-of-treatment radiotherapy. In a retrospective study of 303 patients with stage I/II NK/T-cell lymphoma, sequential chemotherapy and radiotherapy gave CR, PFS and OS that were comparable with those of concurrent chemoradiotherapy with or without subsequent consolidation chemotherapy [64]. The use of SMILE (dexamethasone, methotrexate, ifosfamide, L-asparaginase and etoposide) [65], GELOX (gemcitabine, L-asparaginase, and oxaloplatin) [66,67], P-GEMOX (pegaspargase, gemcitabine, and oxaliplatin) [68], and MESA (methotrexate, etoposide, dexamethasone, and pegaspargase) [69], followed by or sandwiched with radiotherapy, also gave ORRs of 90–100%, and CRs of 74–91%, which resulted in 5-year PFS of 64–83% and OS of 64–90%. These excellent results depend critically on the use of asparaginase-containing regimens. In a large retrospective analysis of more than 2000 patients with early stage NK/T-cell lymphoma, the 5-year PFS and OS of patients receiving sequential radiotherapy and anthracycline-based regimens were poor at 54.4% and 65.3% [70]. In contrast, patients receiving non-anthracycline-based regimens had significantly superior 5-year PFS and OS of 67.7% and 77% [70]. In the same cohort (N = 376), the use of asparaginase-based chemotherapy regimens was associated with a significantly better 5-year OS (hazard ratio: 0.55), as compared with that of non-asparaginase based regimens when they were given sequentially with radiotherapy [71]. These results have underscored the importance of choosing the optimal chemotherapeutic regimens to be used with radiotherapy. Furthermore, these data also indicate that anthracycline-based regimens are inferior for NK/T-cell lymphoma and should be abandoned.

#### 9.1.3. Radiotherapy

Radiotherapy as the sole modality in stage I/II NK/T-cell lymphoma is associated with unacceptably high failure rates. Therefore, radiotherapy is only considered an adjunct to chemotherapy. Incorporating radiotherapy in the treatment algorithm of stage I/II NK/T-cell lymphoma has been reported to improve outcome, particularly when the chemotherapy regimens used were not intensive [72,73]. The dose of radiotherapy should be at least 50Gy, in order to decrease in-field relapse [74,75]. Intensity modulated radiation therapy (IMRT) provides adequate tumour target coverage with sparing of normal surrounding tissues and is widely used [76]. In a retrospective study comparing the use of IMRT and three-dimensional conformal radiotherapy (3D-CRT) for early stage NK/T-cell lymphoma, patients receiving IMRT with or without chemotherapy had a superior 5-year PFS (69%) and OS (76%) as compared with those receiving 3D-CRT (5-year PFS and OS were 58% and 68%, respectively) [77]. Hence, radiotherapy is merely part of the treatment in NK/T-cell lymphoma and should be used alone only in patients too frail to tolerate any chemotherapy [59].

### 9.2. Management of Stage III/IV NK/T-Cell Lymphoma

The mainstay of treatment for stage III/IV NK/T-cell lymphomas is combination chemotherapy that incorporates asparaginase in the regimens [9]. Similar to stage I/II disease, anthracycline-based regimens have been shown to be ineffective and should not be used [70]. Regimens incorporating L-asparaginase or pegylated asparaginase including SMILE [65,78], AspaMetDex (L-asparaginase, methotrexate, and dexamethasone) [79], MEDA (methotrexate, etoposide, dexamethasone and pegylated asparaginase) [80] and P-GEMOX [81] have been evaluated in patients with newly diagnosed advanced-stage NK/T-cell lymphomas. With the SMILE regimen in treatment-naïve stage III/IV patients, 40–54% of cases achieved CR, with a 5-year OS of 47% [65]. In a phase III randomized study comparing DDGP (dexamethasone, gemcitabine, cisplatin and pegaspargase) with SMILE in newly diagnosed stage III/IV patients, the results of DDGP were apparently superior, with a higher CR (71%), and better 1-year PFS (86%) and 1-year OS (90%) [82]. However, the results of the SMILE group in this study was exceptionally poor, with CR of only 29% and a 1 year-OS of 57%; which were inferior to those of the phase II study [78] and the real-world setting [65]. Hence, the significance of this study remained doubtful.

The outcome of stage III/IV NK/T-cell lymphomas remains unfavourable, despite the reasonable CR rates with asparaginase-containing regimens. Therefore, the appropriate use of post-remission strategies in stage III/IV cases is important in order to improve long-term results.

## 10. Haematopoietic Stem Cell Transplantation (HSCT)

Early results of autologous HSCT in 18 patients showed that outcome was poor for patients transplanted not in remission, suggesting that at least some prior degree of disease control must be achieved [83]. These early observations were confirmed in a later retrospective analysis of frontline autologous HSCT in 62 patients. The 3-year PFS and OS were 64.5% and 67.6% for early stage patients, and 40.1% and 52.3% for advanced-stage patients, respectively. Multivariate analysis showed that patients with disease not under control before autologous HSCT had significantly inferior outcome [84]. In a recent phase II study of upfront autologous HSCT after VIDL induction chemotherapy in 27 patients with advanced-stage NK/T-cell lymphoma, 17 patients achieved CR or partial remission after 4 cycles of chemotherapy and underwent HSCT [85]. With a median follow up of 31.2 months, only 8 patients remained in CR after HSCT. It can be seen from these results that frontline HSCT did not appear to be superior to concurrent or sequential chemoradiotherapy for early stage disease and asparaginase-based regimens for advanced-stage disease. Therefore, frontline autologous HSCT is generally not recommended, because of its uncertain additional benefit.

Allogeneic HSCT offers a potential cure for high-risk patients, owing to its putative graft-versus-lymphoma effect. However, there is no randomized prospective study to evaluate its role. In highly selected patients with advanced-stage or relapsed/refractory (R/R) disease, a 5-year OS of more than 50% could be achieved with allogeneic HSCT [86,87]. However, allogeneic HSCT is associated with a high treatment-related mortality and there is a lack of prognostic and predictive markers for identification of high-risk patients who would benefit most from it.

## 11. Practical Recommendations for Management of NK/T-Cell Lymphomas

For stage I/II patients, asparaginase-containing regimens combined with radiotherapy can be considered the standard of care. Sequential chemotherapy and radiotherapy are used in most centres, owing to logistic simplicity. Furthermore, patients often have much better performance at the time of radiotherapy, which has fewer adverse effects when used alone. Concurrent chemoradiotherapy is hardly used, owing to logistic complexity and higher toxicity. Monitoring of plasma EBV DNA should be routinely performed. The aim should be to achieve non-quantifiable plasma EBV DNA after the initial two or three cycles of chemotherapy [49]. At end of-treatment, both EBV DNA and PET/CT should be normal, such that durable remission may be achieved [49,50,53]. Autologous HSCT has no role for stage I/II patients in clinical, molecular and radiologic remission. Regular monitoring of plasma EBV DNA should be undertaken during follow-up.

For stage III/IV patients, asparaginase-containing regimens are the standard. Patients who present with widespread disseminated disease should be carefully monitored for tumour lysis and liver function derangement, which may be related to cytokine released from the neoplastic lesions. Once disease control is achieved, allogeneic HSCT should be considered for suitable patients. CNS prophylaxis is not recommended for stage III/IV patients, because the incidence of CNS involvement or relapse is low. However, for regimens that do not contain intermediate to high dose methotrexate, which is included in SMILE or similar regimens, there appears to be an increased risk of CNS relapse [40]. Whether the use of CNS prophylaxis is justified in patients receiving such treatment should be further validated. Similar to stage I/II patients, the aim must be to achieve clinical, molecular and radiologic remission at end-of-treatment assessment.

## 12. R/R NK/T-Cell Lymphomas

The management of R/R NK/T-cell lymphomas remains challenging. For patients treated with first-line anthracycline-containing regimens, the use of asparaginase-containing regimens will achieve a high remission rate. However, for patients who have failed asparaginase-containing or other non-anthracycline-containing regimens, the outlook is bleak, with a median PFS of 4.1 months and OS of 6.4 months [88]. Therefore, novel treatment modalities are needed for these patients.

### 12.1. Immune Checkpoint Blockade Therapy for R/R NK/T-Cell Lymphomas

Histologic studies have shown overexpression of PD-L1 in NK/T-cell lymphomas, providing a theoretical basis of targeting the PD1/PD-L1 axis in R/R diseases [89]. In the first published series of seven patients treated with the anti-PD1 antibody pembrolizumab after failing asparaginase-based regimens and allogeneic HSCT, all patients responded after a median of seven cycles, with five patients achieving CR [90]. No treatment-related adverse events were observed. In another report of seven patients with R/R disease treated with pembrolizumab, four patients responded to treatment, and two of them achieved CR [91]. A durable response was observed in one patient who remained in CR after 18 cycles. In another 19 patients treated with pembrolizumab, there was no correlation between PD-L1 expression and response to treatment [92]. However, whole genome sequencing identified somatic mutations in the 3′-untranslated region of the PD-L1 gene as a predictive marker for response [92]. The effectiveness of the anti-PD1 antibody nivolumab in R/R NK/T-cell lymphoma has also been described in three patients, all of whom responded, with one case achieving continuous CR after nine cycles [93]. Another anti-PD1 antibody sintilimab was studied in 28 patients with R/R NK/T-cell lymphoma [94]. Although only two patients achieved CR, the ORR was 67.9%, and the 2-year OS was 78.6% [94].

Targeting anti-PD-L1 has also been studied. The use of avelumab, an anti-PD-L1 antibody, was reported in a phase II study of 21 patients [95]. The ORR was 38% and CR was 24%. Five patients have durable response after a median of 18 cycles. PD-L1 expression in lymphoma cells correlated with response [95]. No grade 4 adverse events were observed.

The disparity in responses to PD-1/PD-L1 blockade in these studies may be related to the relatively small sample size, heterogeneity of patient characteristics, and the use of anti-PD1 vs. anti-PD-L1 antibodies. Overall, the efficacy of PD-1/PD-L1 immune blockade therapy is promising for R/R disease, although further studies on the identification of predictive biomarkers for response are needed. The effectiveness of combining a PD1/PD-L1 blockade with chemotherapies or other novel treatment is warranted.

### 12.2. Other Immunotherapies including Cellular Therapy

The antigen CD38 is strongly expressed in about 50% of NK/T-cell lymphomas and has been shown to be a prognostic marker [96]. Anecdotal report of the efficacy of the anti-CD38 antibody daratumumab had been described in R/R NK/T-cell lymphoma [97]. However, in a phase II prospective study of daratumumab monotherapy in 32 patients with R/R NK/T-cell lymphomas, the ORR was merely 25%, with no CR attained [98]. Responses were independent of CD38 expression. With a median follow-up of 10 months, the median PFS and OS were 53 and 141 days only, suggesting that daratumumab monotherapy was not effective [98]. CD30 is expressed in 30–50% of NK/T-cell lymphomas. The anti-CD30 antibody conjugate brentuximab vedotin had been reported to be efficacious in two patients with R/R NK/T-cell lymphomas expressing CD30 [99,100], an observation requiring prospective validation.

Another strategy to harness anti-tumour immunity is adoptive cellular therapy. There has been strong evidence supporting the use of EBV-specific cytotoxic T-lymphocytes (CTL) to target EBV-associated lymphomas [101,102]. In NK/T-cell lymphomas, EBV latency II viral proteins LMP1, LMP2 and EBNA1 are less immunogenic and susceptible to immune surveillance [101]. Production of active autologous EBV-specific CTL targeting these latency type II EBV-positive tumours would require the use of adenoviral vector transduced dendritic cells expressing LMP1 and 2, and EBV-transformed lymphoblastoid cell lines as antigen-presenting cells to activate and expand EBV-antigen specific CTL [102]. In a recent phase II study of 47 patients with R/R NK/T-cell lymphoma, autologous EBV-specific CTL was successfully generated in 32 cases, with 15 patients subsequently infused with the products [103]. Ten of them had active lymphoma at the time of treatment and received a median of four doses of EBV-specific CTL. The ORR was 50%, with a CR rate of 30%. The median PFS was 12.3 months [103]. No grade 3 or 4 adverse events were observed. Cytokine release syndrome and immune effector cell-associated neurotoxicity syndrome were not observed. The high manufacturing failure rate (32%) and the long turnaround time (approximately 25 days), however, limit the clinical utility of this treatment approach.

### 12.3. Novel Agents

Chidamide is an oral selective histone deacetylase (HDAC) inhibitor of HDAC1, 2, 3, and 10 [104]. In two studies with 49 cases of R/R NK/T-cell lymphomas [105,106], salvage treatment with chidamide resulted in an ORR of 18% (CR: 6%). Alisertib, a selective aurora A kinase inhibitor, had been examined in five patients of R/R NK/T-cell lymphomas [107,108], with only one case (20%) achieving a partial response. Other drugs approved in the treatment of peripheral T-cell lymphoma, including romidepsin and pralatrexate [109], had been used in too few cases of NK/T-cell lymphoma for their efficacy to be determined.

## 13. Future Treatment Strategies

Novel treatment approaches are needed for advanced-stage and R/R NK/T-cell lymphoma because outcome with current treatment remains poor. Combination of strategies with different mechanisms of action may improve outcome. In a phase Ib/II trial of chidamide combined with sintilimab, data reported in abstract form showed that, of 45 patients with R/R NK/T-cell lymphomas, 36 cases were evaluable, with an ORR of 58.3% and a CR rate of 44.4% [110], which appeared to be better than that of either agent alone in other studies. An immune checkpoint blockade may also work synergistically with conventional chemotherapy or cellular therapies. With the promising data on anti-PD-1 or anti-PD-L1, other immune blockade therapies are also worth studying. LAG3 and TIM-3 are expressed in more than 90% of NK/T-cell lymphomas, suggesting that antibodies against these T-cell inhibitory molecules might be potential therapeutic agents [111]. Furthermore, advances in the understanding of the tumour biology of NK/T-cell lymphoma have identified putative driver molecules or pathways such as the JAK-STAT pathway as potential druggable targets for therapeutic intervention.

## 14. Conclusions

NK/T-cell lymphomas are rare malignancies with specific clinicopathologic features and treatment strategies. Anthracycline-containing regimens for conventional lymphomas are ineffective. Non-anthracycline regimens containing asparaginase are the standard. Stage I/II lymphomas are treated with combined radiotherapy and chemotherapy. Stage III/IV lymphomas are treated with chemotherapy. Novel approaches including checkpoint inhibitors are needed for relapsed/refractory lymphomas.
